# Intra-rater reliability of determining positions of cervical spinous processes and measuring their relative distances: An update to define rigid bodies of the cervical spine in a movement laboratory setting

**DOI:** 10.1186/s13104-019-4299-8

**Published:** 2019-05-14

**Authors:** Markus J. Ernst, Bettina B. Sommer, André Meichtry, Christoph M. Bauer

**Affiliations:** Institute of Physiotherapy, Zurich University of Applied Sciences, Technikumstrasse 71, 8401 Winterthur, Switzerland

**Keywords:** Bony landmarks, Spinous process, Cervical spine, Reliability

## Abstract

**Objectives:**

A reliable detection of bony landmarks of the spine is necessary in order to determine rigid bodies and to reduce the variability of marker placement in a movement laboratory setting. In a first study on the thoracic and lumbar spine, we demonstrated that placing markers on their relative positions between two major landmarks was superior to palpation of specific bony landmarks. The aims of this study were to examine the intra-rater reliability when palpating for spinous processes (SPs) of the second (C2) and seventh cervical vertebrae (C7), to determine the distances between C2 and C7 and the relative position of C7 along the length between C2 and the posterior superior iliac spine (PSIS) level.

**Results:**

The intra-rater reliability in determining the distance between C2 and C7 was found to be substantial, with an intra-rater reliability of 0.75 (95% confidence limits 0.55–0.99) and a standard error of the measurement of 0.34 cm. The relative distance of C7 along the total C2–PSIS length was estimated to be 11.5%. The determination of the relative positions of spinal landmarks through measurement is considered superior to their palpation, because it relies on a reproducible and comparable definition of rigid bodies.

## Introduction

Systematic reviews that focus on the palpation of bony landmarks of the spine claim that palpation is only reliable and valid in determining dysfunctional spinal segments when additional information, such as pain or tenderness at the sites, is available [1, 2]. This information may be useful for direct intervention, such as manual treatment of the painful site [3, 4], but in a movement laboratory setting the reliable detection of the bony landmarks of the spine, such as spinous processes (SPs), is most important in order to reduce variability in marker or sensor placement (for interrater or intersession settings). These bony landmarks are typically chosen to determine rigid bodies, such as the cervical, thoracic or lumbar spine segments, and curvature. In our first study on the thoracic and lumbar spine, we demonstrated that the placement of spinal markers/sensors relative to two major landmarks was more reliable than palpation of specific bony landmarks [5]. An equivalent method to determine the rigid bodies of the cervical spine, such as the upper and lower cervical spine, has not yet been found. In a recent systematic review, by Povoa et al., on the validity of finding bony landmarks of the cervical spine, only one of the five studies included defined cervical landmarks other than C7 [6]. In that study, by Gadotti and Magee, palpation of the SPs of C2, C4, C6 and C7 were validated using radiographs [7]. They showed an overall agreement of 87.8% between palpation and radiographic evaluation. The least error rate was found for C2, which can be easily palpated “as the first bump” while moving downwards from the occiput [7]. In contrast, C4 and C7 showed the largest error rates. Based on their results, the aims of this study were to examine the intra-rater reliability when palpating for SPs of the second (C2) and seventh cervical vertebrae (C7). Further to determine the distances between C2 and C7 and between C2 and the posterior superior iliac spine (PSIS) level and to determine the relative position of C7 along the total C2–PSIS length.

## Main text

### Methods

**Fig. 1:**
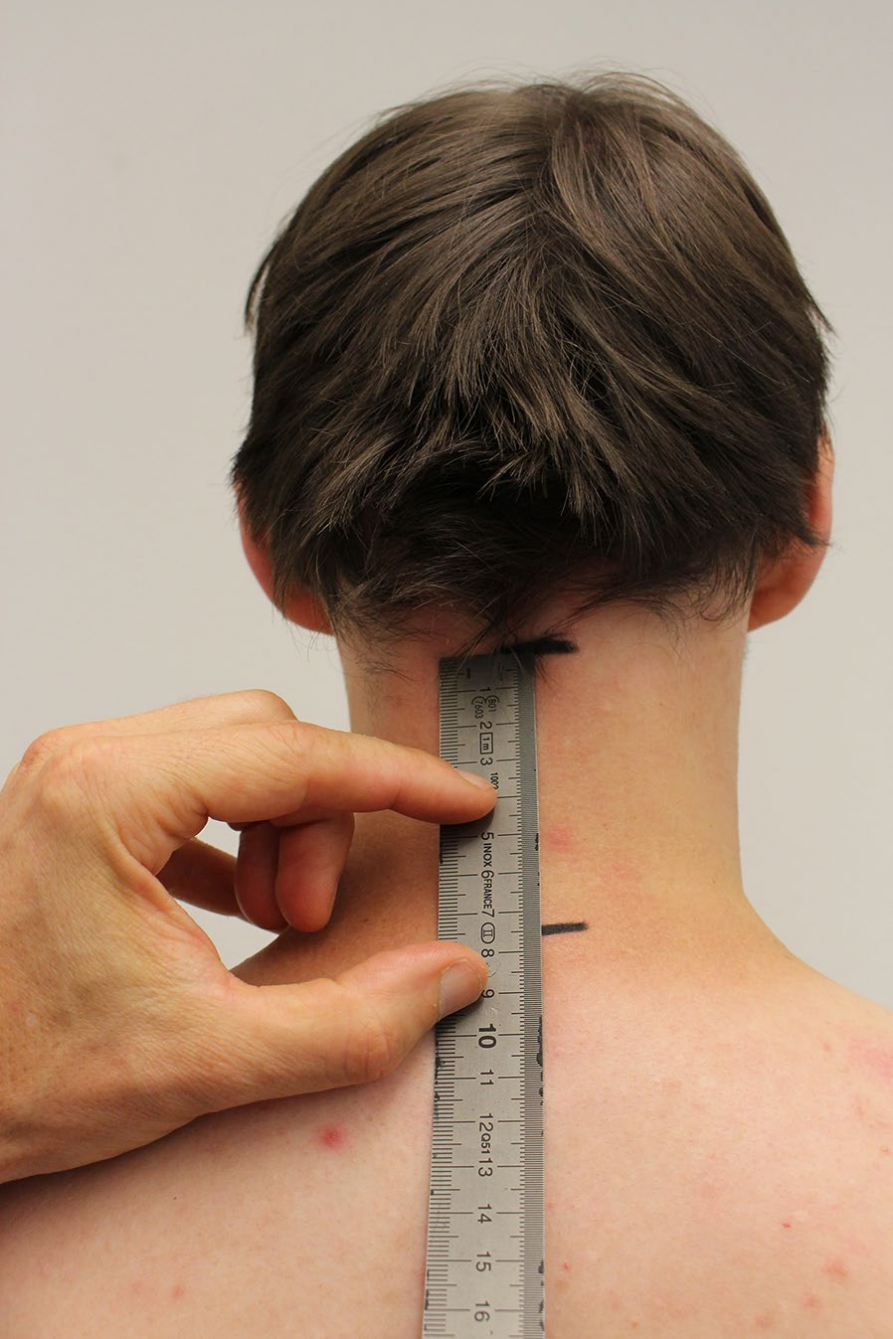
Measuring the distance between C2 and C7 spinous processes markings using a flexible ruler

This study used a repeated-measures design. A consecutive sample of asymptomatic subjects was tested, who were recruited for another study, determining test–retest reliability of cervical spine movement tests, at the university campus by online advertising. The sample size was based on that study. The guideline for reporting reliability and agreement studies (GRRAS) has been followed [8]. Subjects were included if they were between 18 and 65 years of age, had a body mass index (BMI) between 18 and 28 and were not suffering from an acute disease, specifically musculoskeletal, cardiovascular, neurological and otolaryngological diseases. Subjects were excluded if they had undergone spinal surgery or if they were experiencing bodily pain exceeding an intensity of two, out of a maximum of 10, on a numeric rating scale [9]. The palpation of the cervical bony landmarks was performed by one of three raters (two physiotherapists and one movement scientist, a selected sample of movement laboratory staff), with 5 to 20 years of experience in accurate palpation of bony landmarks including the cervical spine. Spinal processes of C2 and C7 and both posterior superior iliac spines (PSISs) were identified with the subject in a free upright standing position and according to established methods, as described elsewhere [5, 10–12]. The SP of C2 has been detected by moving the hand centrally down from the occiput, while holding the head of the subject into slight extension to relax the dorsal muscles. The first prominent bony landmark detected by this method has been regarded as the SP C2. The three landmarks (SP C2 and C7 and the PSISs level) were marked with a pen and the distances between them measured using a flexible ruler (Fig. 1). The same procedure was repeated 6 to 8 days later at roughly the same time and by the same rater, who has been blinded to their first measurements. Therefore, each rater palpated the same subsample of subjects twice, independently from the other raters. Due to their training and experience, the three raters were regarded members of a similar population, warranting an intra-rater reliability study design. Since the study was part of a larger study, the procedures resembled conditions similar to a daily movement laboratory routine.

### Data analysis

The generalizability theory [13] with the design S(R) × R × T, Subjects (nested in rater) × rater × time was used as a framework to estimate reliability of bony cervical spine landmark palpation, based on the linear model:

$${\text{Y}}_{\text{i j k}} = \upmu + {\text{ S}}\left( {\text{R}} \right)_{\text{i}} + {\text{ R}}_{\text{j}} + {\text{ T}}_{\text{k}} + {\text{RT}}_{\text{i k}} + \upepsilon_{\text{i j k}}$$

with μ representing the global mean, S(R)_i_ corresponds to S_i_ + SR_i j_ which cannot be disentangled in a nested design and ε_i j k_ the independent and normal distributed errors ε_i_ ~ N(0, σ^2^).

Intra-rater reliability was calculated as G coefficient:

${\text{G }}\left( {{\text{Y}}_{{{\text{i }}\left( {\text{j}} \right),{\text{k}}}} , {\text{ Y}}_{{{\text{i }}\left( {\text{j}} \right),{\text{k}}^{{\prime }} }} } \right)\, = \,\frac{{\sigma^{2} S\left( R \right)}}{{\sigma^{2} S\left( R \right) + \sigma^{2} T + \sigma^{2} RT + \sigma^{2} \varepsilon }}$, with σ^2^ being the variance of subjects (nested in rater), rater, time and ε_i j k ._

A G coefficient of < 0.2 demonstrates slight, 0.2–0.39 fair, 0.4–0.59 moderate, 0.6–0.79 substantial and > 0.8 almost perfect reliability between measurements [14].

Additionally the standard error of measurement (SEM) was computed by using the formula: SEM = σ_y_ $\sqrt {1 - G}$ with σ_x_ being the standard deviation of the observed scores and G the established G coefficient [15].

In addition, the total C7–PSIS distance was determined through measurement, as in our first study [5]. Finally, the relative percentage position of SP C7 along the C2–PSIS length was calculated.

### Results

Twenty of 23 subjects were eligible for inclusion: 7 male/13 female, mean age of 35.4 years (SD 12.6), mean BMI of 22.6 kg/m^2^ (SD 2.4). Three subjects were excluded as they scored too high on BMI (n = 1) or NDI (n = 1) or missed the second measurement (n = 1). The average distance measured between the spinous processes C2 and C7 was 6.52 cm (SD: 0.88). A substantial intra-rater reliability of 0.75 was found with a SEM of 0.34 cm (Table 1).

**Table 1:** Intra-rater reliability: distance C2 to C7 in centimetres (n = 20)

1st rating mean (SD)	2nd rating mean (SD)	Mean (SD) of both ratings	95% CI	G-coefficient (95% CI)	SEM
6.60 (1.07)	6.45 (0.79)	6.52 (0.88)	± 0.41	0.75 (0.55–0.99)	0.34

*G-coefficient* intra-rater reliability coefficient, *95% CI* 95% confidence interval, *SEM* standard error of the measurement

The relative percentage position of SP C7 along the SP C2–PSIS length was at 11.5% (95% confidence interval 10.8–12.2%) (Table 2).

**Table 2:** Percentage position on the length from C2 to PSIS

Variable	Value (%)	Lower limit (%)	Upper limit (%)
C2	0		
C7	11.5	10.8	12.2
PSIS	100		

Values are means, limits are 95% confidence limits according to a t-distribution with 19 degrees of freedom; C2-PSIS = distance between spinal process of the second cervical vertebrae and the posterior superior iliac spine; C7 = spinous process of the seventh cervical vertebrae

### Discussion

This study demonstrates a substantial reliability for the palpation of the spinous processes of C2 and C7 and the measurement of the intervening distance in asymptomatic subjects when performed by one rater. The position of the SP C7 was shown to be at 11.5% of the total distance between the SP C2 and the PSIS level.

The reliability value found in this study (0.75) is slightly lower than in our first study, in which Intraclass correlation coefficients (ICCs) were ≥ 0.967, even for inter-rater reliability, which is commonly supposed to be lower than intra-rater reliability [5, 15, 16]. In the first study, reliability was determined through measuring the distances between SPs with a flexible ruler, and not by palpation [5]. Two raters had to reach agreement on the position of each SP and mark these; measurement of the intervening distances was then performed independently [5]. Furthermore, in the current study we palpated and measured on two time points 6 to 8 days apart, so variations within subjects might also attribute to reliability. However as the time interval has been kept short and both measurements have been taken at the same daytime, we considered this as negligible. We regard the demonstrated intra-rater reliability of 0.75 in this study as acceptable, and the SEM of 0.34 cm is comparable to the findings of our first study (0.2–0.3 cm) [5].

In this study, palpation of the landmarks T4, T7, T10, L1 and L4 has not been conducted. However, their relative positions can be estimated based on the newly determined length of C2–PSIS (C2–C7 plus C7–PSIS).

Palpation of the C2 and C7 SPs and the PSIS (on both sides) was performed by one of three raters and repeated by the same rater after 6 to 8 days. The palpation of SP C7 and PSIS were done according to established criteria [5, 10–12, 17, 18]. A recent study by Ferreira et al. found better criterion validity for the location of SP C7, when compared to radiographs, by detecting the first rib, following this posteriorly to the SP of the first thoracic vertebrae and then moving upwards, which is supposed to be C7 [19]. This method has been found more accurate compared to the commonly used “Flexion–Extension Method” used in this study, in which the SP C7 remains stationary and the SP C6 moves forward with passive extension of the neck [12, 19]. However, criterion validity for both methods remains very low, with 18% for the Flexion–Extension method and 33% for the new “following the first rib” method [19]. Further information of the subject such as age, body mass index and the distance from SP C7 to the vertex at the cranium in centimetres could even better predict the exact location of SP C7 in 66% of the cases in the study by Ferreira et al. [20].

The detection of the SP C2 as the “first bump” while palpating down from the occiput has shown far better criterion validity, with less than 2% misclassification [7], whilst the palpation of SP C7 seems to be more error-prone. This may be because it differs less from the adjacent SPs of C6 and T1, or because of its main anatomical differentiation (a divided SP for C6, but not for C7, is not easily determinable with soft tissues such as the nuchal or supraspinous ligament overlying the SPs) [21]. We regard this to be a supporting argument for palpation of SP C2 instead of defining its position relative to C7. Reliability of palpation and distance measurements can be further improved by standardisation, such as where to exactly draw the line on the skin over the SP, at the upper, middle or lower end; and to instruct the subject to “push back” during drawing that line and measuring the distance.

## Limitations

We regard our findings valid for subjects with no major spinal deviations, especially in the sagittal plane. The sample size was small (n = 20), with only seven male subjects. Misclassification of the C2 and C7 SPs could have occurred, even though palpation was performed according to the aforementioned criteria. We have only considered intra-rater reliability, so it is possible that values could vary with different raters and different time-points. Radiographic confirmation of palpation was not considered feasible within the framework of this study, but may be advisable for future studies. Since the length measurements were performed in a free upright standing position, it is possible that the head and neck could have been pushed forward by the rater when applying the flexible ruler. This could have led to differences between the repeated measurements. Although each subject was asked to report any “pushing forward movement”, some might have been unaware of this. Therefore, for future studies, a supported and upright position while leaning the forehead against a wall could be a better measurement stance.

## Data Availability

The datasets used and/or analysed during the current study are available from the corresponding author on reasonable request.

## Abbreviations

BMI: body mass index
CI: confidence interval
ICC: intra-class correlation coefficient
PSIS: posterior superior iliac spine
SP: spinous process
SEM: standard error of the measurement
SD: standard deviation
